# HO-SsNF: heap optimizer-based self-systematized neural fuzzy approach for cervical cancer classification using pap smear images

**DOI:** 10.3389/fonc.2024.1264611

**Published:** 2024-05-01

**Authors:** Ashok Shanmugam, Kavitha KVN, Prianka Ramachandran Radhabai, Senthilnathan Natarajan, Agbotiname Lucky Imoize, Stephen Ojo, Thomas I. Nathaniel

**Affiliations:** ^1^ Department of Electronics and Communication Engineering, Vel Tech Multi Tech Dr. Rangarajan Dr. Sakunthala Engineering College, Chennai, Tamil Nadu, India; ^2^ Department of Communication Engineering, School of Electronics Engineering, Vellore Institute of Technology, Vellore, Tamil Nadu, India; ^3^ Department of Artificial Intelligence and Machine Learning (AIML) New Horizon College of Engineering, Chennai, Tamil Nadu, India; ^4^ Department of Design and Automation, School of Mechanical Engineering, Vellore Institute of Technology, Vellore, Tamil Nadu, India; ^5^ Department of Electrical and Electronics Engineering, Faculty of Engineering, University of Lagos, Akoka, Lagos, Nigeria; ^6^ Department of Electrical and Computer Engineering, College of Engineering, Anderson University, Anderson, IN, United States; ^7^ School of Medicine Greenville, University of South Carolina, Greenville, SC, United States

**Keywords:** cervical cancer, classification, optimization, GLCM, LBP

## Abstract

Cervical cancer is a significant concern for women, necessitating early detection and precise treatment. Conventional cytological methods often fall short in early diagnosis. The proposed innovative Heap Optimizer-based Self-Systematized Neural Fuzzy (HO-SsNF) method offers a viable solution. It utilizes HO-based segmentation, extracting features via Gray-Level Co-Occurrence Matrix (GLCM) and Local Binary Pattern (LBP). The proposed SsNF-based classifier achieves an impressive 99.6% accuracy in classifying cervical cancer cells, using the Herlev Pap Smear database. Comparative analyses underscore its superiority, establishing it as a valuable tool for precise cervical cancer detection. This algorithm has been seamlessly integrated into cervical cancer diagnosis centers, accessible through smartphone applications, with minimal resource demands. The resulting insights provide a foundation for advancing cancer prevention methods.

## 1 Introduction

The unchecked exponential development phase of cells, the majority of which is carcinoma *in situ* (CIS) or have migrated to the rest of the body, is known as cancer (1). One of the leading causes of cancer-related mortality in women globally is ovarian cancer, a frequent epithelial malignancy (2). Cervical cancer is a frequent malignancy in women all over the world. The most frequent malignancy in women under 35 is cervical cancer, which, if detected early, is curable (3). Early-onset sexual activity, Sexually Transmitted Diseases (STDs), and smoking are associated with an increased risk of developing cervical cancer. Normal squamous cells (NS), aberrant or premalignant cells, and eventually invasive disease are the stages that lead to cervical cancer (4). HPV is identified in cervical cells in many females with cervix dysplasia. HPV infection is widespread in males and females, with sexual intercourse among females under 30 most affected (5). The Papanicolaou (Pap) smear is an excellent approach for detecting precancerous cells via cytological testing for cervical cell anomalies (6). The Pap smear is the most widely used procedure for early cervical cancer screening and diagnosis. On the other hand, the manual analysis of Pap smears is prone to errors owing to human error. Furthermore, the procedure is time-consuming and tiresome.

The percentage of squamous epithelium that fails to mature at the epithelium’s interface is termed low-grade dysplasia (LGD)/CIN I, high-grade dysplasia (HGD)/CIN II, as well as CIN III and additional CIS (7). Early detection and diagnosis of this cancer form are crucial for effective treatment. However, automated cell separation from cervical smears faces challenges due to noisy backgrounds, weak cytoplasmic contrast, and fuzzy, overlapping cells, making it a significant hurdle in biomedical image processing. Therefore, the development of a computer-assisted assessment tool to enhance the accuracy and reliability of the Pap smear test is valuable. Various computer-aided diagnostics (CAD) methods for cervical cancer screening rely on engineering features such as artificial neural networks (ANN) (8), quasi-supervised learning (QSL) (9), K-nearest neighbor (KNN) models (10), and Support Vector Machines (SVM) (11) have yielded significant results for binary and multiclass problems, with SVM garnering substantial research attention (12).

Adjuvant vaccination plays a crucial role in bolstering the immune response against specific diseases, particularly in cases where primary treatment might not offer complete protection. In the context of high-grade cervical intraepithelial neoplasia (CIN2+) and early-stage cervical cancer, adjuvant vaccination post-hysterectomy could potentially target residual or recurrent disease, offering a secondary line of defense against human papillomavirus (HPV) infections. This approach may help reduce the risk of disease recurrence and progression, improving the overall prognosis for patients. Furthermore, adjuvant vaccination could contribute to broader public health goals by reducing the transmission of HPV, thus potentially lowering the incidence of cervical cancer in the population.Researchers are currently exploring the impact of vaccination after hysterectomy for high-grade cervical intraepithelial neoplasia (CIN2+) and early-stage cervical cancer, as existing data is lacking in this area. Additionally, efforts are underway to better categorize patients with human papillomavirus (HPV) to improve prognosis and tailor surveillance strategies accordingly (13, 14).

However, traditional techniques are only suitable for high-resolution cervical images, with suboptimal sensitivity and accuracy rates for broader cervical cancer diagnoses (15). These methods typically identify only the exterior border regions of cancer areas. To overcome these limitations, we introduce a fully automated computer-assisted methodology for cervical cancer area screening in cervical images using an enhanced intelligent hybrid classification approach called HO-SsNF. The primary contributions of this research are summarized as follows:

The research focuses on image capture and employs a common dataset for analysis. The preprocessed cell images are fed into an HO-based segmentation model to extract features from an image.

- Moreover, GLCM and LBP are used to extract the features from the segmented output.
- HO-SsNF is used to classify the segmented images once the appropriate features have been extracted.
- A baseline comparison was constructed for the overall experiment using the pap smear database, which includes the complete cell and allows all characteristics to be retrieved.

The rest of the article is outlined as follows: The recent literature related to this research is provided in Section 2. The problem report of this research is explained in Section 3. The proposed methodology is described in Section 4. The results and comparison are discussed in Section 5, and Section 6 concludes the work.

## 2 Related work

Several recent works relevant to this research can be summarized as follows:

Sellamuthu et al. (16) proposed a modified deep learning system for the detection and categorization of Pap smear cell images, a crucial step in cervical cancer diagnosis. Their approach relied on a dual-tree complex wavelet transform (DTCWT) and convolutional neural networks (CNN) to categorize these images into four distinct classes: normal, cancer *in situ*, dysplastic, and superficial. While this method showed promise, it was noted to have a significant computational time requirement.

Adhikary, Shreya et al. (17) tackled the differential interference contrast (DIC) dataset, employing classification techniques such as multilayer perceptron (MLP), SVM, and k-NN after cell segmentation using a modified valley-linked Otsu’s threshold approach. Principal component analysis (PCA) was also utilized to select features and enhance classifier performance.

Nuclei segmentation faces challenges due to uneven staining, complex backgrounds, and overlapping cell clusters, which impact image quality. Zhao, Meng et al. (18) proposed a novel Nuclei Segmentation method based on Selective-Edge-Enhancement (SEENS). This approach divided whole-slide cervical images into smaller regions of interest, reducing repeated segmentation and eliminating non-nuclei areas.

Chen, Hua, et al. (19) introduced CytoBrain, an artificial intelligence (AI) system designed to filter abnormal cervical cells to aid in subsequent diagnostic procedures. CytoBrain consisted of three core components: cervical cell identification, cell classification, and a human-aided diagnostic unit. Their findings highlighted the productivity and efficiency of the CompactVGG-based method in large-scale cervical cancer screening.

Yaman, Orhan, and Turker Tuncer (20) proposed the use of a pyramid-based deep feature extraction approach for cervical cancer detection, focusing on classifying cervical cells in Pap smear images. They utilized Neighborhood Component Analysis (NCA) to derive interesting and insightful features.

Ghoneim et al. (21) introduced a cervical cancer cell pattern identification system based on a convolutional neural network (CNN), followed by classification using an extreme learning machine (ELM). Transfer learning and fine-tuning were employed in the CNN architecture. Their CNN-ELM-based system achieved a 91.2% classification accuracy for a 7-class problem, suggesting potential performance improvement with additional filtering in convolutional layers.

Ozbay, E., and zbay, F.A et al. (22) developed a method for tumor image retrieval in the cervical cavity using hash coding and a Convolutional Neural Network (CNN). They proposed a deep hashing technique incorporating mask synthesis and rotation invariance to detect cervical cancer.

Desiani, A (23). proposed two pathways combining image segmentation and classification. They enhanced images using techniques such as Normalisation, CLAHE, and Adaptive Gamma Correction before segmentation, aiming to improve picture quality. The first pathway employed CNN-based segmentation, while the second utilized KNN and ANN algorithms for classification.

Chauhan, N.K. et al. (24) suggested a hybridization of a deep feature concatenated network (HDFCN) with two data augmentation steps for cervical cancer identification. They employed a combination of features from pretrained deep learning (DL) algorithms to categorize cancerous specimens from Pap smear images.

Jeyshri, J. and Kowsigan, M (25) presented a method to divide multiclass cells into the nucleus and cytoplasmic regions using a multi-resolution U-Net (MRU-Net) for medical image segmentation. This approach aimed to overcome limitations associated with U-convolution Net-based kernels and ill-defined ideal system width, potentially improving cervical cell abnormality detection by physicians.

Mishra, A.K. et al. (26) described technique called Brightness Maintaining Dynamic Fuzzy Histogram Equalization for image enhancement utilizes the fuzzy c-means technique for element identification and region of interest selection. Subsequently, feature selection using the Ant Colony Optimization (ACO) algorithm is employed. For classification, the technique utilizes Multilayer Perceptron (MLP), Convolutional Neural Network (CNN), and Artificial Neural Network (ANN) techniques.

Kavitha R. et al. (27) developed a computerized Cervical Precancerous Lesion Classification system using Quantum Invasive Weed Optimization with Deep Learning (CPLC-QIWODL) based on biological Pap smear images. Their approach involved preprocessing images with Gabor filtering (GF) and employing the deep variational autoencoder (DVAE) algorithm for classification, achieving a maximum detection rate of 99.07%.

The above set of research papers fillup the research gap in cervical cancer care disparities globally with our method to improvise the testing methods. The summary of the literature review is provided in **Table 1**.

**Table 1:** Related works.

References	Year	Contribution	Findings	Shortcomings
Ozbay, E. and zbay, F.A et al. (22)	2023	CNN	Eliminated unimportant aspects	These techniques will result in numerous collisions
Desiani, A (23).	2023	CNN, KNN, and ANN	The quality of images is improved, and analyzed the value of the metric	Accuracy needs to improve for better segmentation.
Chauhan, N.K., et al. (24)	2023	HDFCN	The suggested model had good accuracy	The CNN-based DL models can cause data duplication
Jeyshri, J. and Kowsigan, M (25)	2023	MRU-Net	Accuracy is 89% only obtained	Performance measure shows poor diagnosis function.
Mishra, A.K. et al. (26)	2023	fuzzy c-means, ACO algorithm, MLP, CNN, and ANN	Precision and Dice are less than 89%	Accuracy is very lower than the other methods
Kavitha, R. et al. (27)	2023	CPLC-QIWODL and DVAE	the maximum detection rate of 99.07%. is achieved	Convergence speed is less for detection
Yaman, Orhan, and Turker Tuncer (20)	2022	NCA and SVM	Best accuracies for both datasets	It has the dataset, and there is no extrinsic validation
Sellamuthu et al. (16)	2021	DTCWT and CNN (ResNet 18)	The image classification is 99%.	The computational time is quite complicated.
Adhikary, Shreya, et al. (17)	2021	MLP, SVM, and k-NN, PCA	Accuracy is 97 and 93% for 2 and 3-class issues	for precise segmentation and are unable to separate entire nuclei from DIC pictures, the quality of this calculation should be improved
Zhao, Meng, et al. (18)	2021	SEENS	High precision value is achieved	Only nuclear characteristics are taken into consideration
Chen, Hua, et al. (19)	2021	CompactVGG	A simple and effective approach for single-cell image extraction.	In order to get the desired result, the applicator selection criteria must be improved.
Ghoneim et al. (21)	2020	CNN-ELM	Without utilizing any characteristics created by hand, outstanding accuracy was attained.	An additional filtering method is required for performance improvement.

Compared to conventional cytological methods, which often fall short in early diagnosis, this method offers a promising solution. The integration of this algorithm into cervical cancer diagnosis centers, accessible through smartphone applications, with minimal resource demands, enhances its accessibility and utility in clinical settings (28). This study contributes to the existing literature by demonstrating the effectiveness of the HO-SsNF method and highlighting its potential as a valuable tool for precise cervical cancer detection. Future research can build upon these findings to further improve early detection methods and advance cancer prevention strategies.

## 3 Proposed methodology

The automated system for computer-aided cervical cancer identification is designed to streamline the process and improve accuracy. It begins by preprocessing raw cervical images, which involves various steps such as noise reduction, contrast enhancement, and image normalization to ensure optimal image quality. The next step involves applying a median filter image enhancement technique specifically tailored for cervical images. This technique helps to further enhance the image quality by reducing noise and sharpening edges, which is crucial for accurate feature extraction.

**Figure 1:**
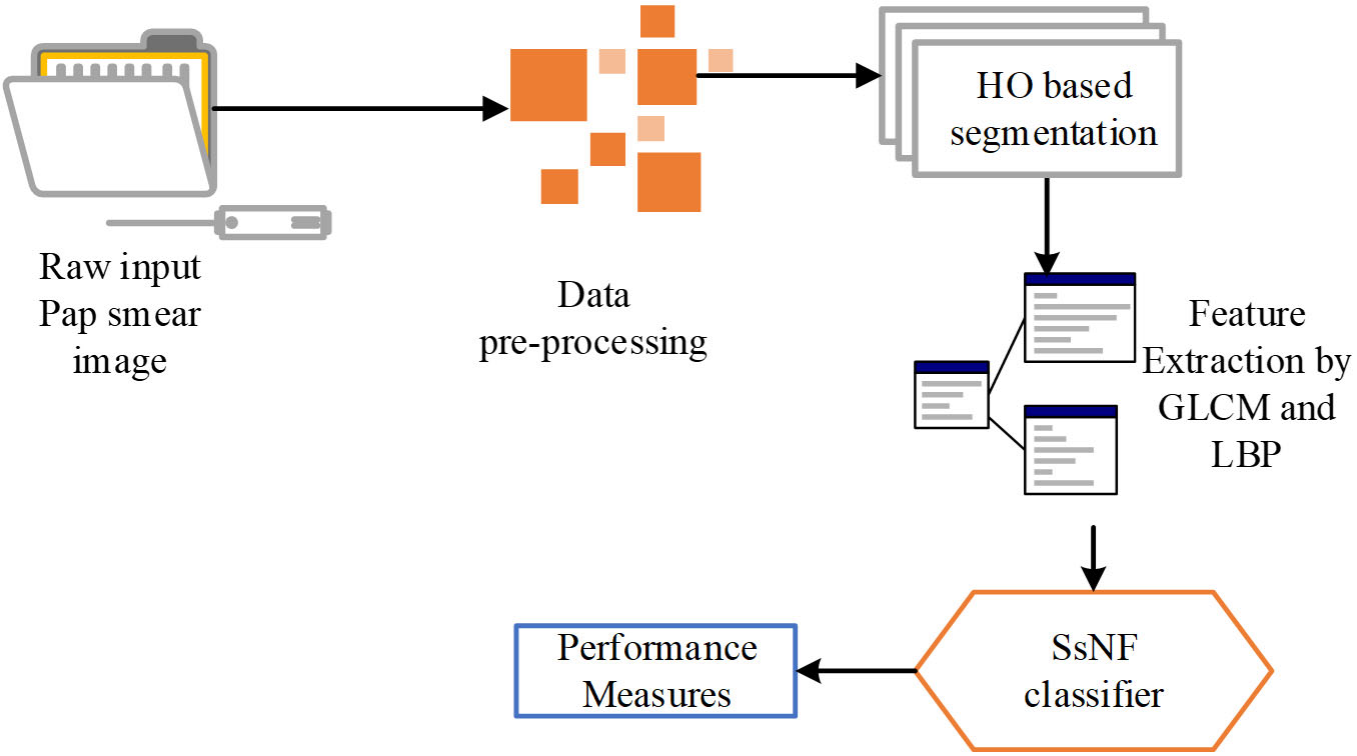
Proposed system methodology.

Following image enhancement, the preprocessed images undergo attribute extraction using a higher-order (HO) based segmentation technique. This technique extracts a variety of features from the images, including texture features like Grey-Level Co-occurrence Matrix (GLCM) and Local Binary Patterns (LBP), which are known to be informative for cervical cancer identification. These extracted features serve as the input for the SsNF classifier, a sophisticated machine learning model trained to classify cervical images as either normal or carcinoma based on the extracted features. The SsNF classifier utilizes a combination of supervised learning techniques to learn the relationship between the extracted features and the corresponding cervical cancer status. This allows the classifier to accurately classify new, unseen images based on their extracted features. Finally, the system evaluates the performance of the classification using standard metrics such as sensitivity, specificity, and accuracy, providing valuable feedback on the effectiveness of the automated system. Overall, this system represents a comprehensive approach to automating cervical cancer identification, leveraging advanced image processing and machine learning techniques to achieve high accuracy and efficiency. The proposed system methodology is as shown in **Figure 1**.

### 3.1 Preprocessing

Data preparation is a crucial step in data analysis, involving the transformation of raw, unprocessed data into a format suitable for analysis. This process includes several key steps: first, the analysis of feature values to understand their distribution and characteristics. Next, the selection of relevant feature subsets, which involves identifying and retaining only the most important features for analysis while discarding irrelevant or redundant ones. Finally, the handling of incomplete data, which may involve imputation techniques to fill in missing values or the exclusion of incomplete records. These preprocessing processes are essential for ensuring that the data is clean, consistent, and ready for analysis, ultimately leading to more accurate and reliable results.

### 3.2 HO-based segmentation

After preprocessing the raw data, the image segmentation function is used to classify diseases accurately. Set important parameters such as population size (K) , maximum iterations (P) , number of input variables (G) , and design variable restrictions $\left(V_{i},U_{i}\right)$ . Equation (1) is used to determine the major fitness parameter for the computation,


(1) $$X=\frac{P}{p}$$

This p is the current iteration. Top-down processes and regulations are implemented in a hierarchical organization, and subordinates obey their immediate boss. If each source data is a proximal image to its segmentation reference $d_{i}$ to its source data, this behavior may be replicated by shifting the position of each picture segmentation. A roulette wheel’s goal is to balance these probabilities, which are separated into three parts, such as $o_{1,}o_{2}$ and $o_{3}$ . Equation (2) is updating the extracted pictures,


(2) $$d_{i}^{m}(p+1)=\{\begin{array}{cc} \begin{array}{c} d_{i}^{m}\\ D^{m}+\varphi \beta^{m}\left|D^{m}-d_{i}^{m}(p)\right|\\ N_{i}^{m}+\varphi \beta^{m}\left|N_{i}^{m}-d_{i}^{m}(p)\right|\\ d_{i}^{m}+\varphi \beta^{m}\left|N_{i}^{m}-d_{i}^{m}(p)\right| \end{array} & \begin{array}{c} o\leq o_{1}\\ o>o_{1}\;and\;o\leq o_{2}\\ o>o_{2}\;and\;\;o\leq o_{3}\;and\;\;g({\bar{N}}_{m})<g(d_{i}^{m}(p))\\ o>o_{2}\;and\;\;o\leq o_{3}\;and\;\;g({\bar{N}}_{m})\geq g(d_{i}^{m}(p)) \end{array} \end{array}$$

Where o is a randomly generated number between 0 and 1, m is the vector element’s superscript, is the current iteration, and p are the important arguments. Where the system’s goal function is denoted as φ and β the search agent exploring the image point region $N_{i}^{m}$ if $g({\bar{N}}_{m})<g(d_{i}^{m}(p))$ and only if it supplies a given image value, else it explores another image value $d_{i}^{m}$ in the source data. Thus, image points change their scores regularly based on the previously described equations to converge on the best global solution.

In the field of medical image analysis for cervical cancer classification using Pap smear images, the visualization of segmentation outputs overlaid on the ground truth serves as a crucial step in evaluating the performance of an algorithm or model. This process involves comparing the regions identified by the segmentation algorithm (segmentation outputs) with the actual regions of interest (ground truth) as determined by expert annotations. By overlaying the segmentation outputs onto the ground truth, it becomes possible to visually assess the accuracy of the segmentation algorithm. Areas where the segmentation output aligns well with the ground truth indicate successful segmentation, while discrepancies highlight areas where the algorithm may need improvement. This visual comparison helps to understand the strengths and limitations of the segmentation approach and provides insights for further refinement and optimization.

### 3.3 Feature extraction

The dataset contains numerous properties, but only a select few are relevant for this study. To distinguish between normal and diseased cervical images, this study focuses on extracting features like GLCM (Gray-Level Co-Occurrence Matrix) and Local Binary Pattern (LBP) from Gabor-processed cervical images. Specifically, the GLCM features play a crucial role in discerning differences between normal and malignant cervical images based on metrics such as contrast, energy, entropy, and correlation. Details of Gabor filters are provided in **Table 2**.

**Table 2:** Details of Gabor filters.

Parameter	Value	Explanation
Number of Filters	8	Hypothetical number of Gabor filters used in the project for texture analysis.
Orientations	4	Hypothetical number of orientations (0°, 45°, 90°, 135°) used for each Gabor filter.
Usage	Feature Extraction	Gabor filters are employed to extract texture features from cervical images, aiding in the identification of cancerous regions.

Gabor filters are convolved with the cervical images at multiple scales and orientations. This process results in a set of filtered images, each capturing the texture information at a specific scale and orientation. The responses from these filtered images are then used to construct feature vectors for each pixel or region of interest in the original image.

These feature vectors, which encode information about the texture patterns present in the image, are then fed into a machine learning classifier. The classifier learns to differentiate between normal and carcinoma tissues based on the extracted texture features, ultimately enabling automated identification of cervical cancer.

### 3.4 SsNF-based classification

**Figure 2:**
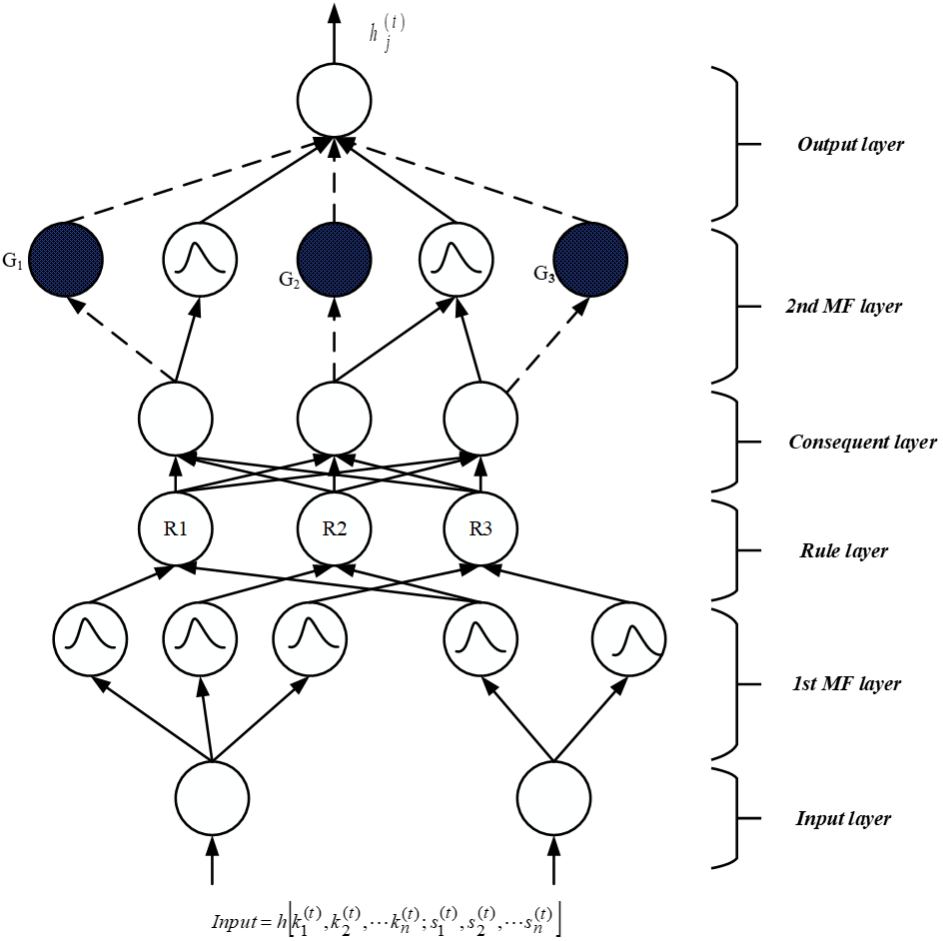
The suggested categorization model.


The SsNF (Self-Systematized Neural Fuzzy) system comprises an integration algorithm connected to a data type fan-in, coupled with information, activation, or evidence from other components. The suggested structure includes four hidden layers, a fuzzification layer, and a defuzzification layer, as depicted in **Figure 2**. During the fuzzification stage, the crisp input obtained from feature selection is transformed into a fuzzy set of values. Equations (3) and (4) are employed to represent the input function of activation and layer result, respectively.

(3) $$Input=h\left[k_{1}^{\left(t\right)},k_{2}^{\left(t\right)},\cdots k_{n}^{\left(t\right)};s_{1}^{\left(t\right)},s_{2}^{\left(t\right)},\cdots s_{n}^{\left(t\right)}\right]$$

where $k_{1}^{\left(t\right)},k_{2}^{\left(t\right)},\cdots k_{n}^{\left(t\right)}$ are the inputs to this component and $s_{1}^{\left(t\right)},s_{2}^{\left(t\right)},\cdots s_{n}^{\left(t\right)}$ the connection weights. The superscript in the above equation indicates the layer number. Each node generates an activation level based on its primary source as its second action.


(4) $$\text{Output}=D_{j}^{\left(t\right)}=G(\text{input})=G(h)$$

Where the activation function is indicated as G(·) . The six classification phases listed below are discussed:

***First input layer:*** No computations are made by this layer. Only input data is transmitted from this layer’s nodes, each of it correlates to a different input parameter. The initial level connection weight ratio $\left[s_{j}^{\left(1\right)}\right]$ is one according to Equation (5), and this is true.


(5) $$h=q_{1}^{\left(t\right)}\;\;\;\;\text{and}\;\;\;\;\;\;G^{\left(1\right)}=h$$

***Second Membership function layer:*** In the initial layer, each unit is associated with one of the input variables for a linguistic parameter. The second layer is responsible for calculating membership functions that define the extent to which input data conforms to fuzzy rules. In this study, a Gaussian membership function is employed, which serves as a global probabilistic model for any nonlinear system, using Equation (6) as its basis.


(6) $$h\left[q_{jx}^{\left(2\right)}\right]=-\frac{{\left[q_{j}^{\left(2\right)}-d_{jx}\right]}^{2}}{\sigma_{jx}^{2}}\text{ and}\;B^{\left(2\right)}(h)=g^{h}$$

where $d_{jx}$ are the mean and $\sigma_{jx}$ is, accordingly, the variance of the $j^{th}$ input parameter’s $q_{j}$ term’s $x^{th}$ Gaussian MF. The weight of a link in this second layer may thus be expressed as $d_{jx}$ .

***Third Rule layer:*** Each node in this tier has one fuzzy inference rule and completes precondition validation. The AND function was used for the third layer element, as shown below is shown in Equation (7):


(7) $$h\left[k_{j}^{\left(3\right)}\right]=\prod_{j}k_{j}^{\left(3\right)}=g^{-{\left[R_{j}(y-d_{i})\right]}^{T}[R_{j}(y-d_{i}]}\;\text{and}\;B^{\left(3\right)}(h)=g^{h}$$

where the number of second layers is marked as engaged in the fuzzy rule’s IF section, and the diagonals are designated as $R_{j}=e\left(1/\sigma_{j1},1/\sigma_{j2},\ldots \ldots 1/\sigma_{jn}\right)$ and $d_{i}=e{\left(d_{i1},d_{i}{\;}_{2},\ldots \ldots d_{i}{\;}_{n}\right)}^{T}$ . The third layer weight link $\left[s_{j}^{\left(3\right)}\right]$ is one. The third layer consequences reflect the firing strength of the related fuzzy rule f .

***Fourth Consequent layer:*** The firing strength produced in the third layer is normalized in this level using Equation (8), and this level has the identical amount of elements as the third layer, and the fourth layer weight link is similarly normalized $\left[s_{j}^{\left(4\right)}\right]$ is one.


(8) $$h[k_{j}^{\left(4\right)}]=\sum_{j}k_{j}^{\left(4\right)}\;and\;G^{\left(4\right)}(h)=\frac{k_{j}^{\left(4\right)}}{g^{h}}$$

***Fifth MF layer:*** The layer under discussion is commonly referred to as the subsequent layer, where two modes are utilized, distinguished by blank and stained circles as depicted in **Figure 2**. Within this layer, the fundamental node represents a fuzzy set characterized by Gaussian membership degrees for the final parameter, denoted by empty circles. In the context of the local mean of maximum (LMOM) defuzzification technique, only the centers of each Gaussian membership value are transmitted to the subsequent layer, while the width primarily contributes to result categorization. To ensure consistent intuitionistic fuzzy values across various rules, multiple fourth-layer terminals can be linked to a single empty fifth-level component. Each shaded element in the fifth layer mirrors a component in the fourth layer, with the output of the fourth layer serving as one of the inputs for a shaded node, alongside the initial layer input variables. The approach for defining a shaded branch, facilitating the creation of a shaded section, can be precisely specified and is illustrated in Equation (9). These two components can be amalgamated to form the fifth layer, which fulfills an overarching function as outlined below.


(9) $$G^{\left(5\right)}(h)=\left(\sum_{j}k_{xj}y_{x}+G_{0j}\right)q_{j}^{5}$$

where $B_{0j}$ is represented as $d_{0j}$ , the Gaussian MF mean. The darkened element $k_{xj}$ is only produced when it is necessary. When the proper variable is supplied, the keywords related to the colored node are added together.

***Sixth output layer:*** This layer’s nodes each represent a unique outcome variable which is shown in Equation (10). The node functions as a defuzzifier, identifying the accurate outcomes, and gathers all of the fifth layer ideas.


(10) $$h\left[k_{j}^{\left(6\right)}\right]=\sum_{j}k_{j}^{\left(6\right)}\;and\;G^{\left(6\right)}(h)=h$$

**Figure 3:**
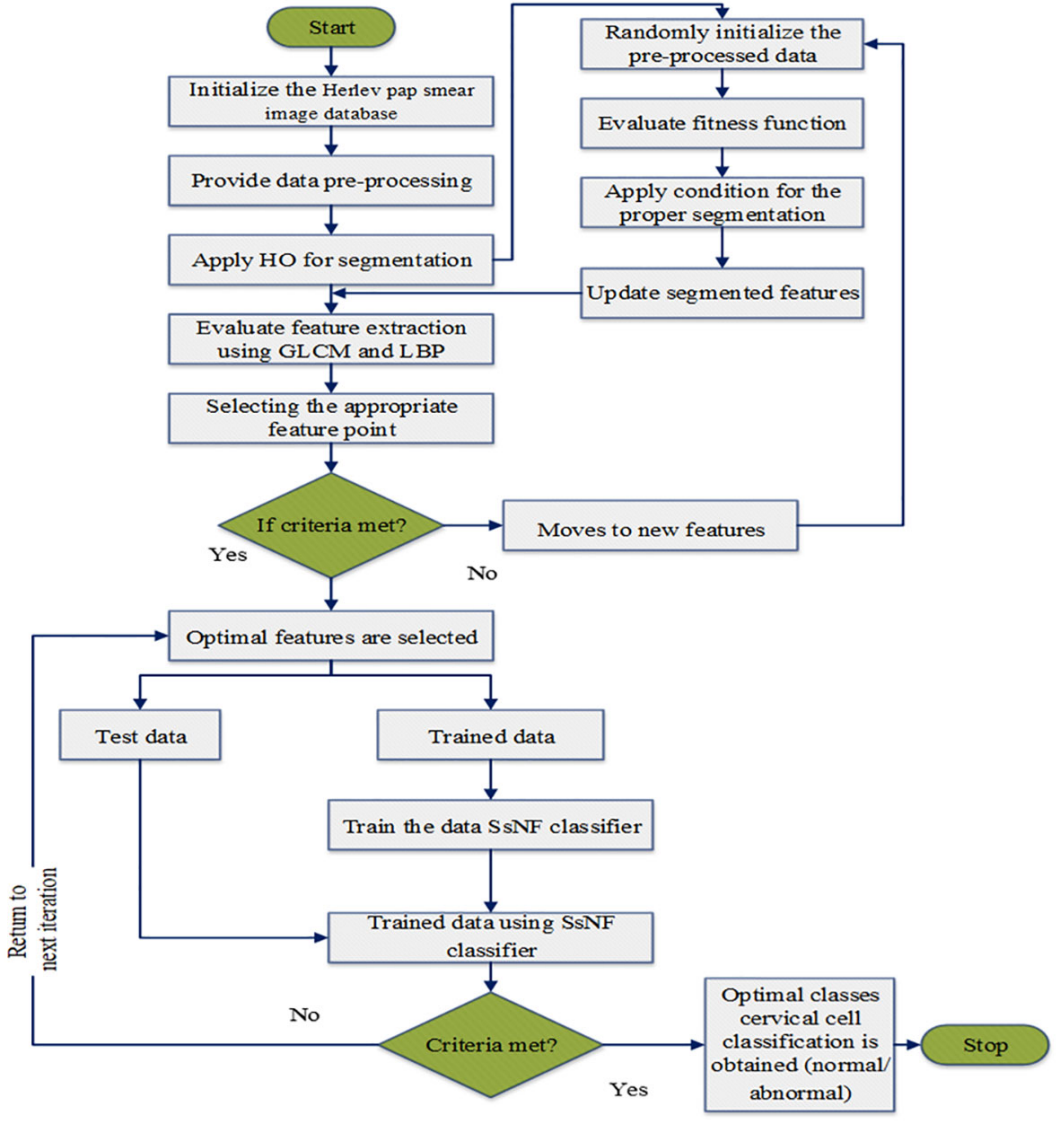
The flowchart of the suggested large data classification algorithm.

The flowchart of the proposed model in big data classification is illustrated in **Figure 3**.

**Fuzzy membership rules are as follows;**

- If the input image exhibits features indicative of normal cervical cells, then the fuzzy membership value for the class “normal” is high.
- If the input image exhibits features indicative of benign cervical cells, then the fuzzy membership value for the class “benign” is high.
- If the input image exhibits features indicative of malignant cervical cells, then the fuzzy membership value for the class “malignant” is high.
- If the input image exhibits features that are ambiguous or not clearly indicative of any specific class, then the fuzzy membership values for all classes are moderate or low.

## 4 Results and discussions

In this section, we present the results of our medical image segmentation efforts and provide an in-depth discussion of the approaches we have proposed. Our study employed MATLAB 2018b on a Windows-based platform for implementation. To assess the proficiency of our proposed approach in classifying medical images, we conducted a comparative analysis between the performance of our developed model and that of conventional methods.

### 4.1 Performance analysis

The efforts made to implement the proposed cervical cancer screening method are appreciated. It makes use of a confusion matrix with dimensions 2x2 and estimates the parameters for True Negative ( ${\vec{\text{T}}}_{\vec{\text{N}}}$ ), False Negative ( ${\vec{\text{F}}}_{\vec{\text{N}}}$ ), True Positive ( ${\vec{\text{T}}}_{\vec{\text{P}}}$ ), and False Positive ( ${\vec{\text{F}}}_{\vec{\text{P}}}$ ) based on regression coefficients from images obtained from a specialized physician. The typical performance measurements include specificity, accuracy, positive predictive value (PPV), recall, sensitivity in Equation (12), and precision in Equation (14).

**Specificity:** It’s a percentage of correctly categorized negative samples. Specificity is shown in (11).


(11) $$\text{S}pecificity\;=\;\frac{{\vec{\text{T}}}_{\vec{\text{N}}}}{{\vec{\text{T}}}_{\vec{\text{N}}}\;+\;{\vec{\text{F}}}_{\vec{\text{P}}}}$$

**Sensitivity/recall:** It measures the percentage of correctly classified positive samples. Sensitivity is a number that varies from 0 to 1.


(12) $$\text{Sensitivity/Recall }=\;\frac{{\vec{\text{T}}}_{\vec{\text{P}}}}{{\vec{\text{T}}}_{\vec{\text{P}}}\;+\;{\vec{\text{F}}}_{\vec{\text{N}}}}$$

**Accuracy:** The amount of successfully categorized snaps determines a method’s accuracy of Classification (i.e., TN as well as TP). Accuracy is shown in Equation (13).


(13) $$\text{Accuracy }=\;\frac{{\vec{\text{T}}}_{\vec{\text{P}}}+{\vec{\text{T}}}_{\vec{\text{N}}}}{{\vec{\text{T}}}_{\vec{\text{P}}}+{\vec{\text{T}}}_{\vec{\text{N}}}+{\vec{\text{F}}}_{\vec{\text{P}}}+{\vec{\text{F}}}_{\vec{\text{N}}}}$$

**Precision/PPV:** It is a metric for image segmentation accuracy and is expressed as a percentage. It is based on the evaluation standards for TP and FP. Precision/PPV is shown in Equation (14).


(14) $$\text{Precision/PPV}=\frac{{\vec{\text{T}}}_{\vec{\text{P}}}}{\vec{\text{T}}{\;}_{\vec{\text{P}}}+{\vec{\text{F}}}_{\vec{\text{P}}}}$$

**NPV:** It records the number of cancer area pixels that were wrongly identified as negative pixels. NPV is shown in Equation (15).


(15) $$\text{N}PV=\frac{{\vec{\text{T}}}_{\vec{\text{N}}}}{\vec{\text{T}}{\;}_{\vec{\text{N}}}+{\vec{\text{F}}}_{\vec{\text{N}}}}$$

### 4.2 Experimental results

The primary objective of this study is to employ advanced deep-learning techniques for the categorization of tumors into different stages. A range of assessment metrics have been applied to gauge the performance of the proposed system. Furthermore, the outcomes generated by our devised approach have been compared with those of various conventional methods utilized in this investigation. The proposed method has undergone rigorous testing using medical datasets from the Herlev Pap Smear Database. The sizes and segmentation details of the cervical cell datasets are outlined in **Table 3**. It is important to note that this research relies on standard Pap smear data, encompassing a total of 1090 images, consisting of 299 normal images and 791 images depicting anomalous stages.

**Table 3:** Sizes and segmentation of cervical cell datasets.

No.	Class term	Number of images	Training	Testing	Validation
1	Intermediate Squamous	78	52 (102)	31	15
2	Normal columnar	89	49 (345)	42	17
3	Normal squamous	132	68 (604)	38	32
4	High-grade dysplasia	433	260 (1,321)	79	49
5	Low-grade dysplasia	129	98 (564)	52	32
6	Moderate dysplasia	124	85(106)	38	15
7	Carcinoma *in situ*	105	102 (405)	41	37
	Total	1090	714 (3,447)	321	197

**Figure 4:**
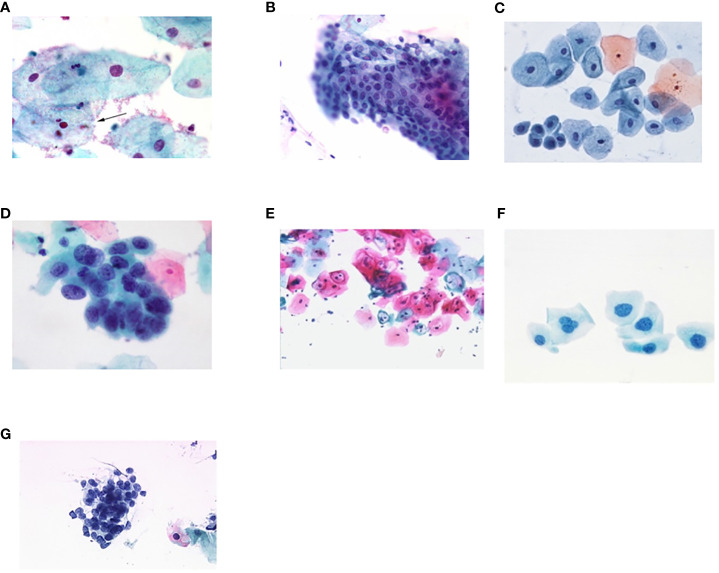
Sample classes of cervical cell pictures **(A)** Intermediate Squamous, **(B)** normal columnar, **(C)** normal squamous, **(D)** HGD, **(E)** LGD, **(F)** moderate dysplasia, and **(G)** Carcinoma *in situ*.

**Figures 4A–G** shows some example images taken from the data set for adequate test cases. Here, the ample classes of cervical cell pictures (a) intermediate squamous, (b) normal columnar, (c) normal squamous, (d) HGD, (e) LGD, (f) moderate dysplasia, and (g) Carcinoma *in situ* are considered for theoretical validation seven images’ results are shown in this article.

The wavelet transform-based noise reduction approach is used in the first phase of the preprocessing stage. This method removes the noise from the images. After processing the images, the HO-based segmentation method is applied to divide the features of the images. The population of images and specific parameter ranges are given as the input of the HO algorithm. The $o_{1,}o_{2}$ and $o_{3}$ of random values are given in the range of -1 to 1. A few factors contribute to HO’s effective exploitation of its audience. First, search agents can take advantage of the vicinity of their superiors or co-cells when a specific parameter is between -1 and 1. Second, the population can develop toward better solutions as the selection probability of Equation (2) gradually increases. Third, a search agent can only change its position if the new position is superior to the old one. In this way, the cell section is segmented as the output. The suggested technique has achieved accurate medical picture segmentation. To extract features in an image, the segmented cell pictures are loaded into a feature extraction method is applied. In the context of the automated system for computer-aided cervical cancer identification, the GLCM matrix is constructed with distance values of 1 and 2, representing the pixel pair distances considered for co-occurrence calculation. A distance of 1 corresponds to immediate neighboring pixels, capturing fine details in the texture, while a distance of 2 includes pixels that are further apart, providing a more global perspective. Additionally, the GLCM is computed at four orientations: 0°, 45°, 90°, and 135°, capturing texture variations in different directions. These parameters are chosen to extract comprehensive texture features from the cervical images, enabling the system to differentiate between normal and carcinoma images based on their texture characteristics. The GLCM matrix features of contrast, energy, entropy, and correlation, derived from these parameters, play a crucial role in the accurate classification of cervical images.

The pap smear database, which covers the total cell and permits all features to be obtained, was used to create a baseline comparison for the total trial. After the proper features have been recovered, The HO-SsNF methodology, is a comprehensive approach that combines elements from hierarchical optimization, self-organization, and neuro-fuzzy systems to enhance the training and performance of neural networks. In this methodology, the training process begins by creating training patterns without applying fuzzy measures to the membership functions of each input parameter, resulting in an equal number of rules for each input parameter’s fuzzy sets. The actual training then occurs over 50000-time steps, with the HO algorithm fine-tuning the subsequent portions of the network. Following training, the system typically exhibits seven output categories and eight input clusters. The fuzzy rule is applied using a Gaussian membership degree function to categorize errors into small, medium, and large values. Finally, the fuzzy rule output is fed to the subsequent layer-shaded node, with other parameters derived from the input node. This methodology provides a structured and effective approach to training neural networks with fuzzy logic, enabling them to learn complex patterns and relationships in the data. Furthermore, a summation of all variables is given in the next part, and finally, classify the type of diseases. (a) intermediate squamous, (b) normal columnar, (c) normal squamous, (d) HGD, (e) LGD, (f) moderate dysplasia, and (g) Carcinoma *in situ*. **Table 4** shows the obtained segmented health consequences for input medical images using the given methodologies.

**Table 4:** Segmented output of the proposed work.

Cancer cell class type	Class term	Input image	Greyscale	Segmentation
The normal type of cells	Intermediate Squamous	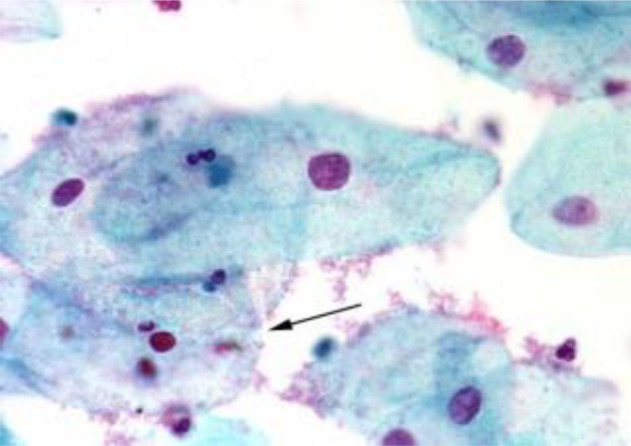	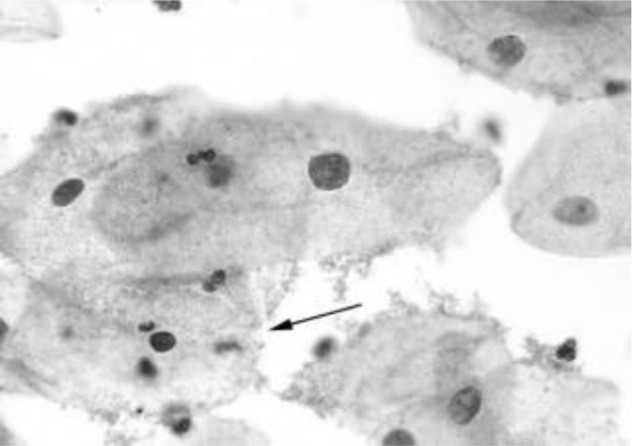	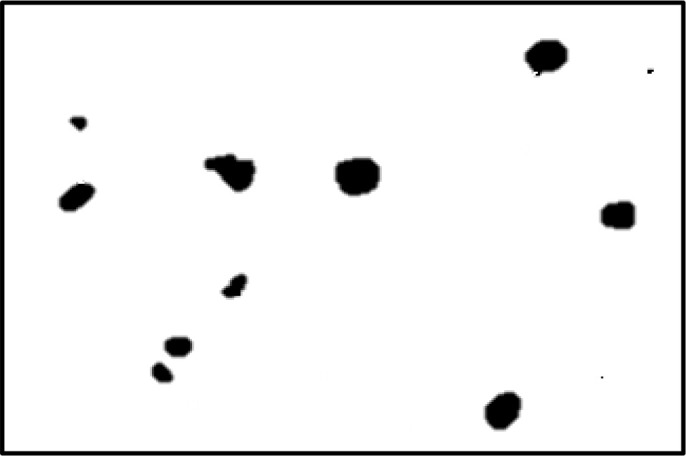
	Normal columnar	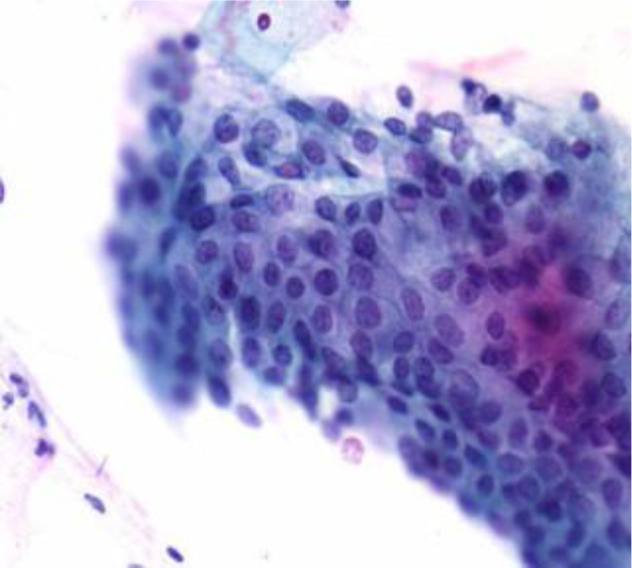	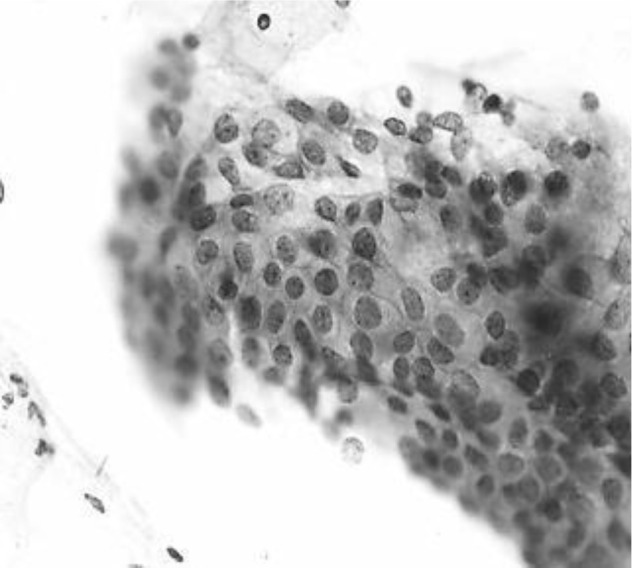	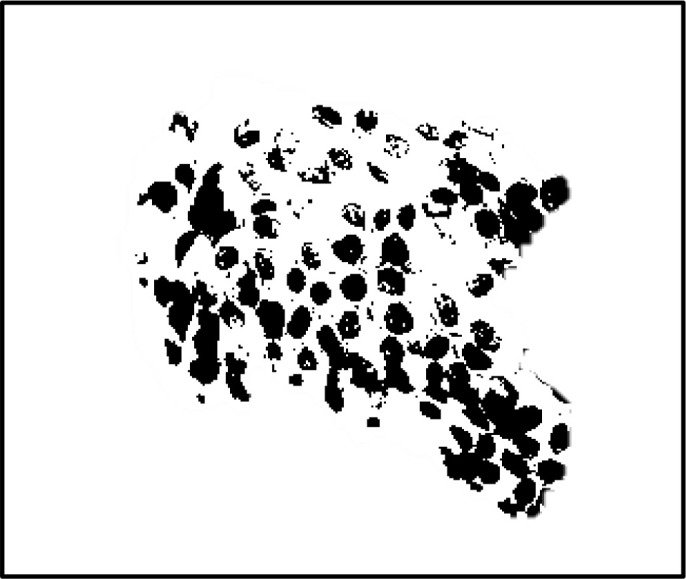
	Normal squamous	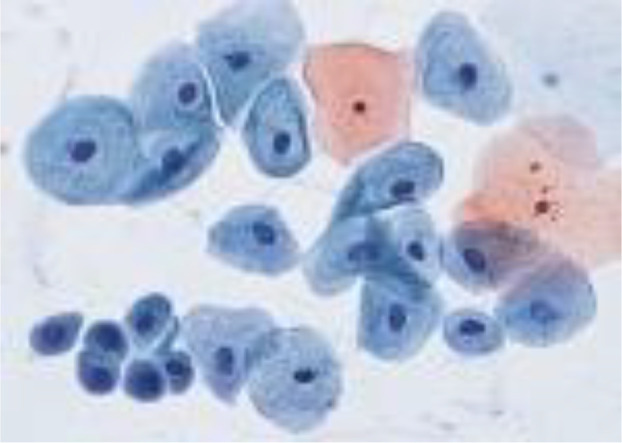	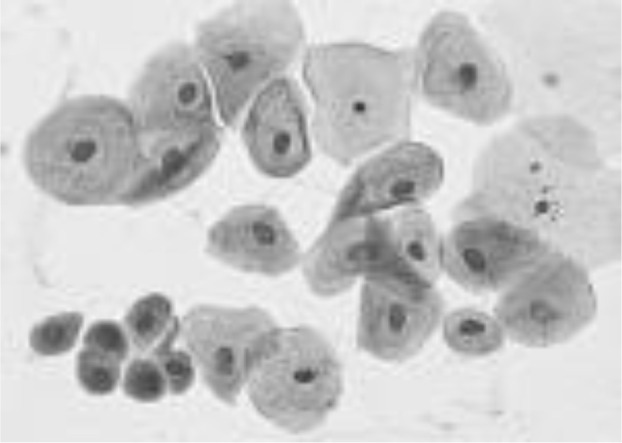	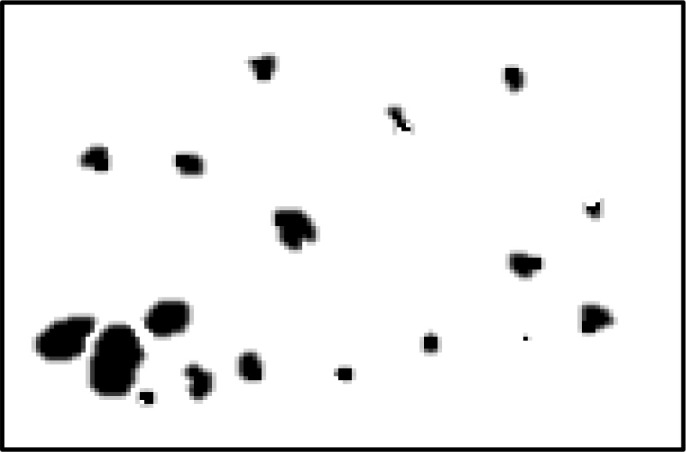
Abnormal type of cells	High grade dysplasia	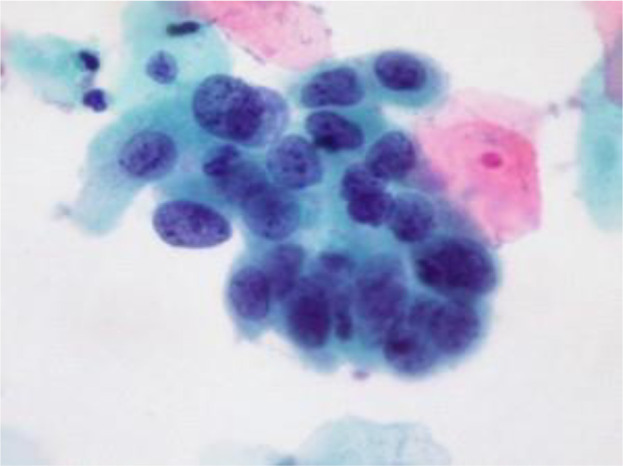	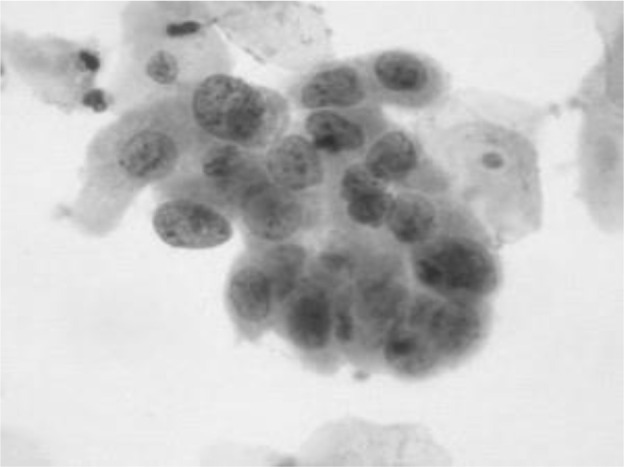	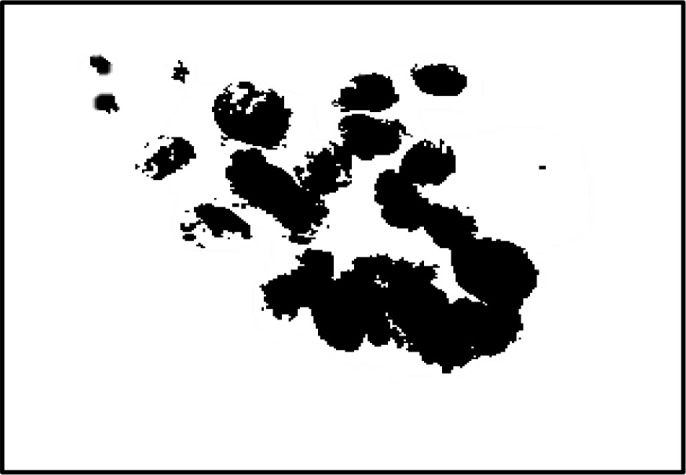
	Low grade dysplasia	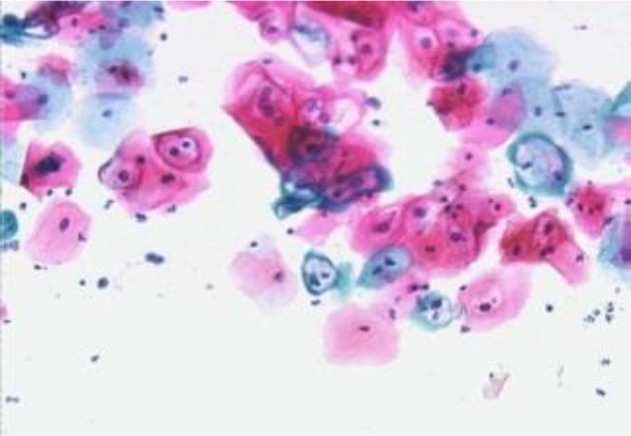	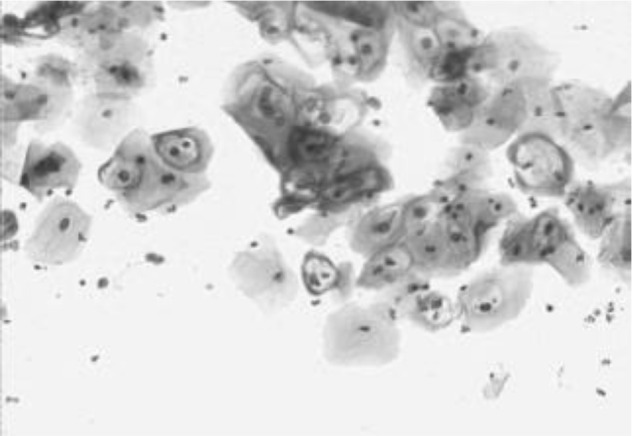	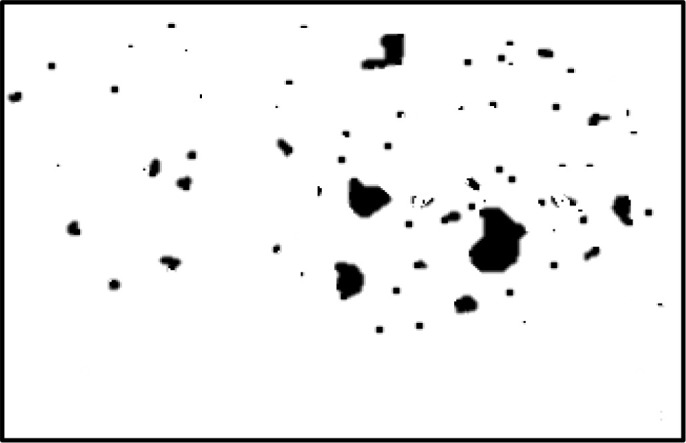
	Moderate dysplasia	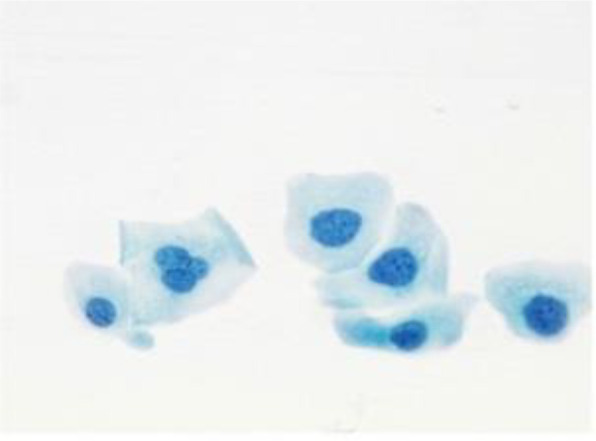	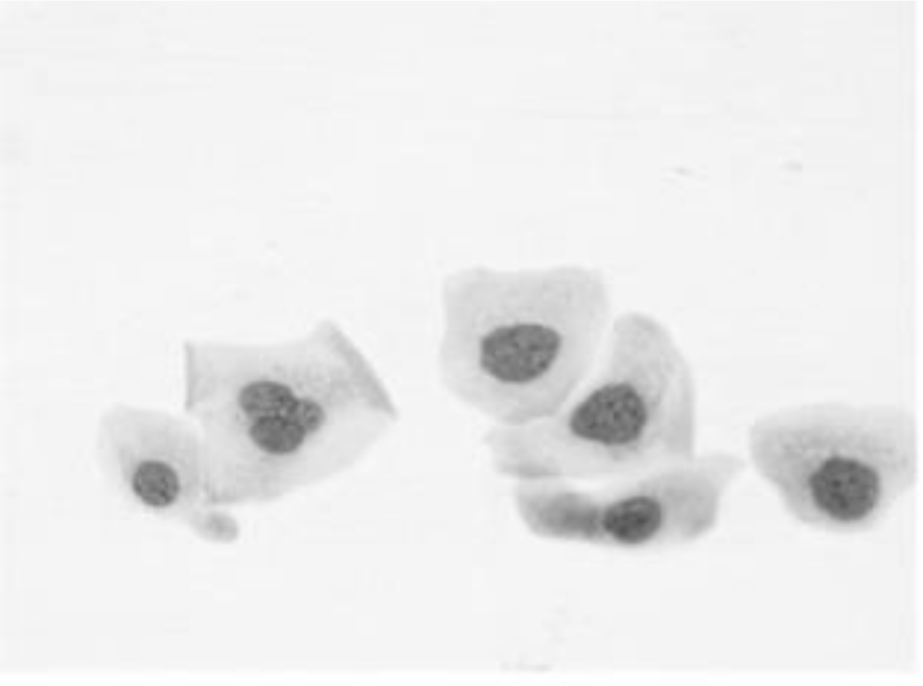	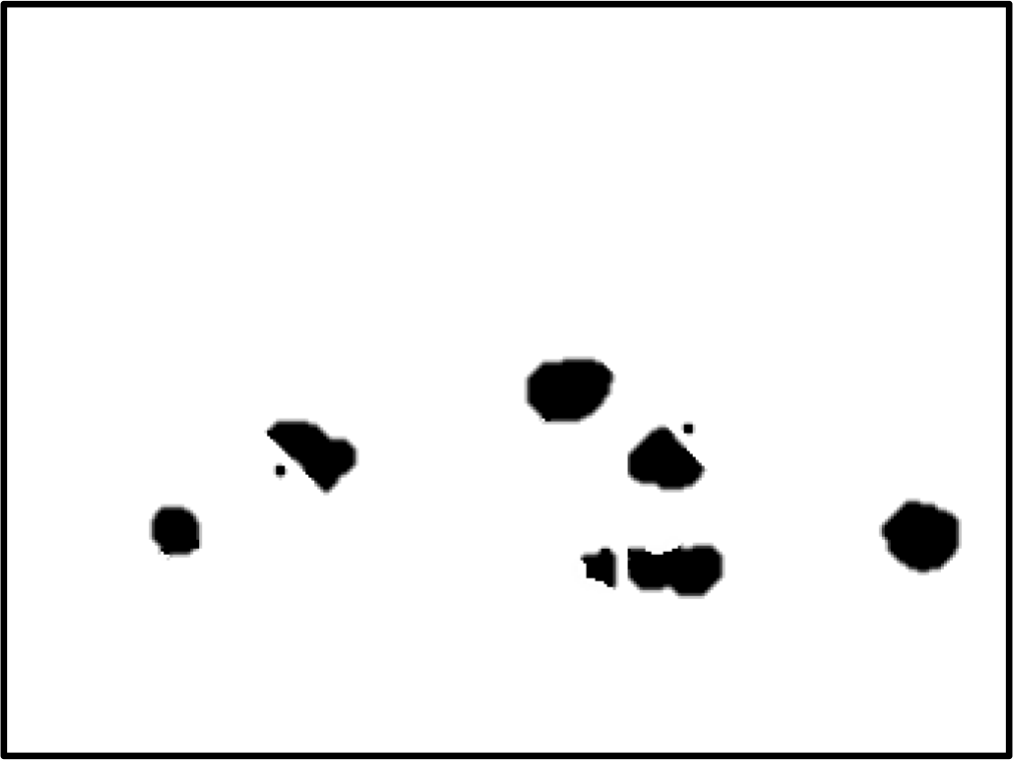
	Carcinoma *in situ*	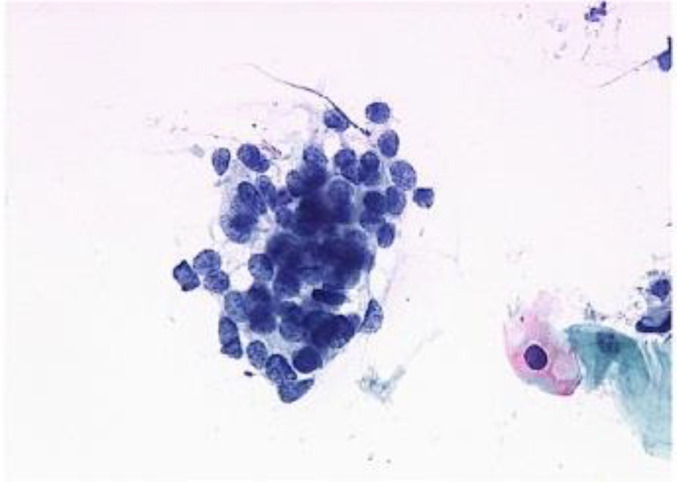	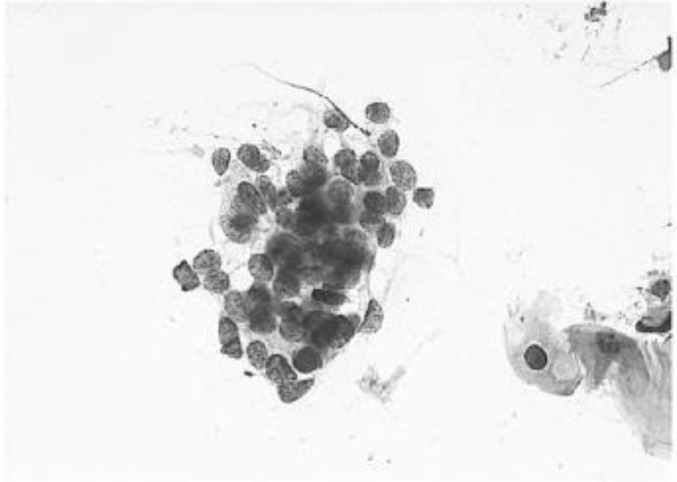	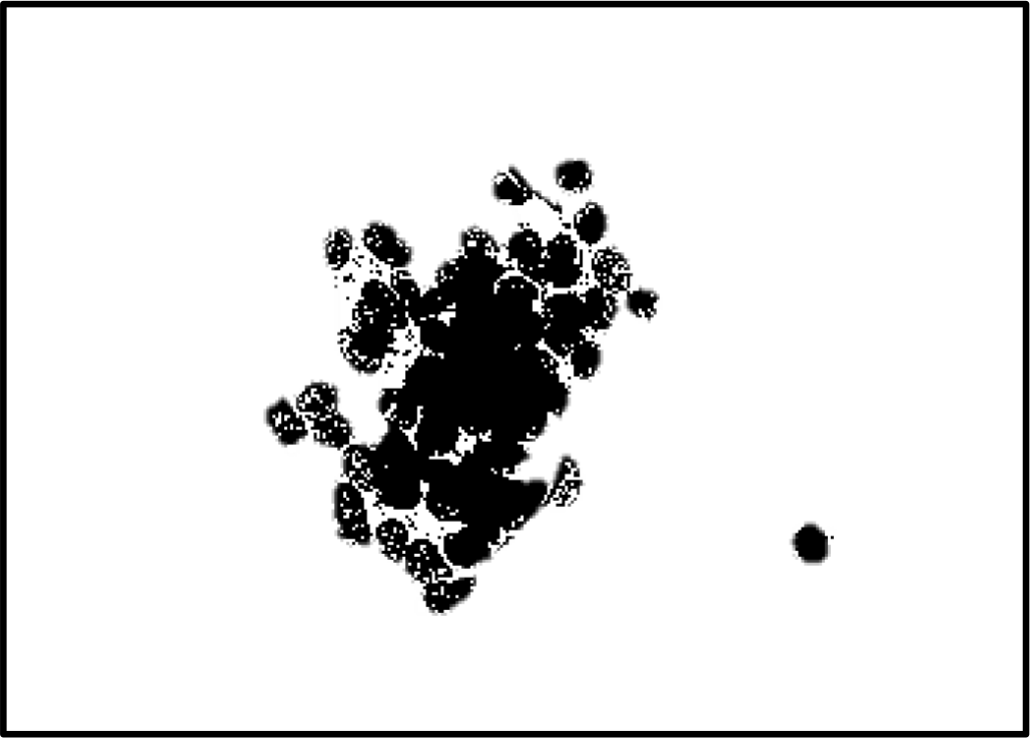

### 4.3 Comparative analysis

**Figure 5:**
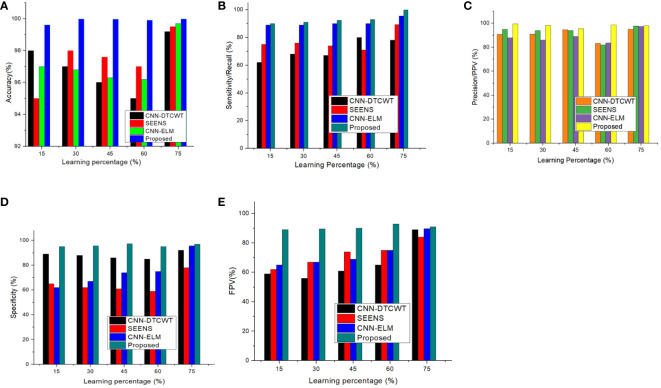
Performance of proposed method and existing method by varying the learning percentage **(A)** Accuracy, **(B)** Sensitivity/Recall, **(C)** Precision/PPV, **(D)** Specificity, and **(E)** NPV.

We have compared the proposed method with other conventional approaches, namely CNN-DTCWT (16), SEENS (18), and CNN-ELM (21), using standard performance evaluation metrics such as Sensitivity/Recall, Accuracy, Precision/Positive Predictive Value (PPV), Specificity, and Negative Predictive Value (NPV). The performance of both the proposed and existing methods was assessed under varying learning percentages, as illustrated in **Figures 5A–E**.

The analysis demonstrates that our proposed method consistently outperforms the previous models in terms of achieving higher values for these performance metrics when the learning percentages are tuned. It’s worth noting that the learning percentage refers to the portion of data used to update the model weights during training. In essence, the learning rate, a hyperparameter, governs the extent to which the model adjusts itself in response to predicted errors.

**Figure 6:**
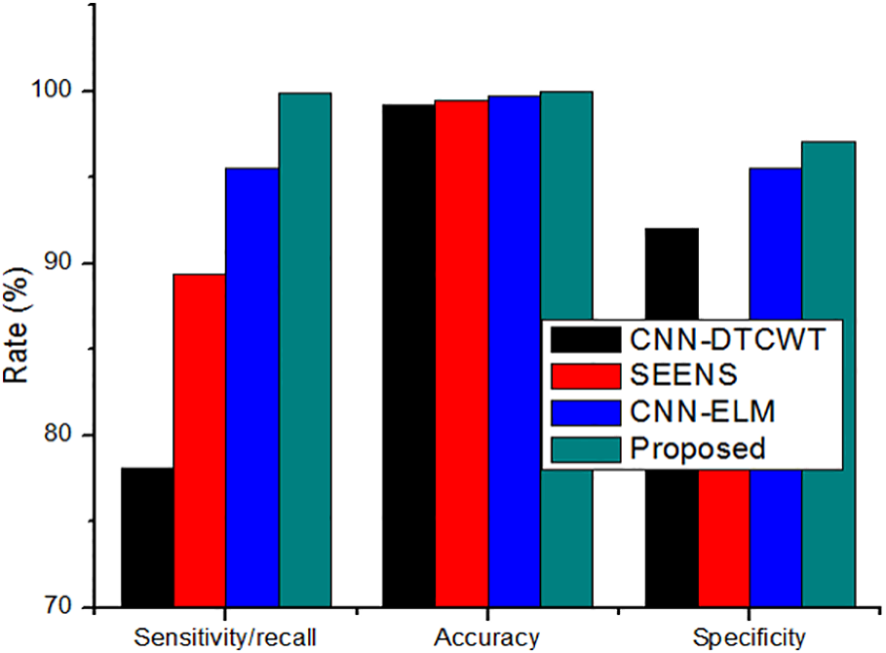
The performance appraisal of segmentation findings of Sensitivity/Recall, accuracy, and specificity.

While increasing the learning percentage, the metrics values are also improved in the proposed method. n addition, **Figure 6** shows the performance appraisal of segmentation findings. The standard CNN-DTCWT has an accuracy of 99.2%, SEENS has an accuracy of 99.50%, and CNN-ELM has an accuracy of 99.7%, which is below the proposed technique of 99.98.

On the other hand, the new method outperformed the existing method, achieving a precision of 0.999. Similarly, the results reveal that the suggested method outperforms previous segmentation strategies regarding sensitivity/recall, precision/PPV, specificity, and NPV.

**Figure 7:**
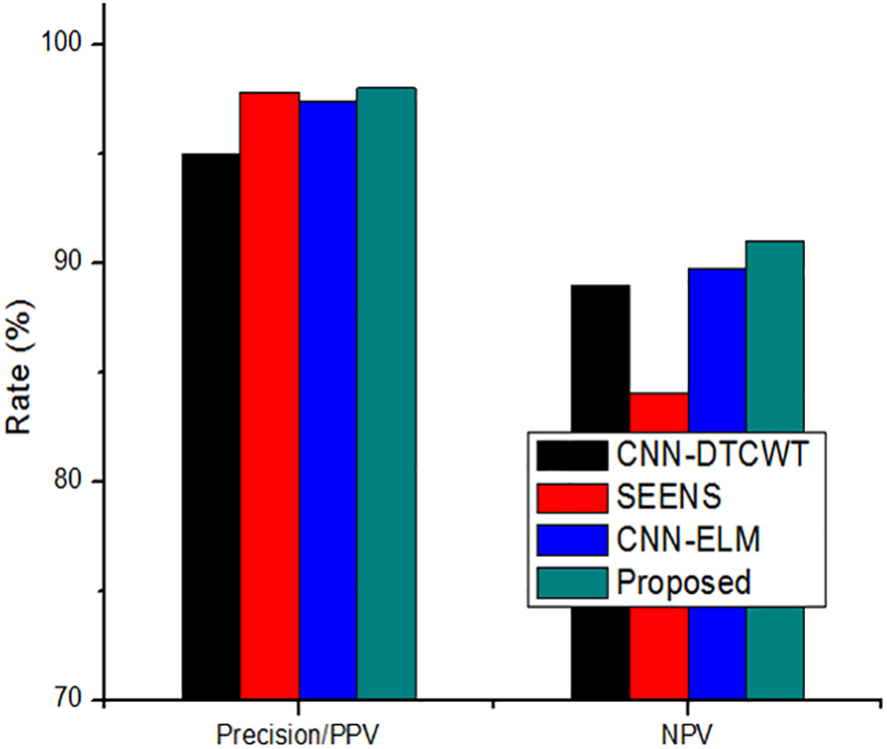
The performance appraisal of segmentation findings of precision/PPV and NPV.

The comparison of the system’s Precision (PPV) and Negative Predictive Value (NPV) to traditional approaches, as depicted in **Figure 7**, highlights the enhanced reliability of the suggested system over previous techniques. This section further delves into the comparison by contrasting the classification results with the most recent approach. **Table 4** serves as a comprehensive platform for these comparisons, demonstrating the superior performance of the suggested strategy over state-of-the-art solutions across various scenarios. In assessing performance, the study employs a range of metrics including accuracy, sensitivity (recall), precision (PPV), specificity, and NPV. Through these comparisons, **Table 5** underscores the superior performance and value of the proposed approach compared to established methodologies, establishing it as a promising advancement in the field of cervical cancer identification.

**Table 5:** Comparative analysis of performance metrics.

Parameters	CNN-DTCWT (16)	SEENS (18)	CNN-ELM (21)	Proposed
Sensitivity/recall	78.09	89.35	95.52	99.89
Precision/PPV	95	97.8	97.42	98
Accuracy	99.2	99.50	99.7	99.98
Specificity	92.01	78.06	95.52	97.1
NPV	89	84.05	89.73	91

### 4.4 Discussions

In the past decade, several models for cervical cancer detection have emerged, exhibiting varying performance based on dataset and methodology. While some conventional methods have proven effective, they rely on manually crafted features and clinical expertise for accuracy. In contrast, deep learning-based approaches have introduced new challenges in medical image segmentation and classification.

To address these challenges, we propose a novel intelligent algorithm for accurate cervical cancer classification. Our results indicate that traditional methods like CNN-DTCWT (16), SEENS (18), and CNN-ELM (21) have limitations compared to our approach. CNN-DTCWT is slower, SEENS requires substantial computational resources, and CNN-ELM exhibits a higher error rate. In contrast, our proposed system offers relatively high reliability, outperforming other image segmentation systems. Although CNN-ELM achieved the highest accuracy, precision, and specificity among prior studies, it falls short of our proposed method’s accuracy. Our technique surpasses SEENS in accuracy, precision, and sensitivity, although it lags behind CNN-DTCWT in certain aspects. Overall, our approach presents a promising solution for accurate cervical cancer classification, bridging the gap between traditional and deep learning-based methods.

In some women with compromised immune systems, reactive oxygen species (ROS) can play a significant role in the development of cervical intraepithelial neoplasia (CIN) and cancer, particularly when combined with high-risk human papillomavirus (HPV) types. ROS are highly reactive molecules that can cause damage to cellular components, including DNA, leading to genetic mutations that contribute to cancer development. When ROS levels are elevated, either due to external factors or internal dysregulation, they can promote the progression of CIN to more severe stages and ultimately to cancer. High-risk HPV types, such as HPV16 and HPV18, are known to be major contributors to the development of cervical cancer. These viruses can infect cervical cells and integrate their DNA into the host genome, disrupting normal cellular processes and promoting uncontrolled cell growth. When ROS levels are high, they can further exacerbate the damage caused by HPV infection, leading to accelerated progression of CIN and increased risk of cancer development. The interplay between ROS and HPV in the development of CIN and cancer highlights the complex nature of these diseases.

Women with poor immune systems, such as those with HIV/AIDS or other immunodeficiencies, may be particularly susceptible to the effects of ROS and HPV, making early detection and intervention critical. Understanding the molecular mechanisms underlying these processes is essential for developing targeted therapies and preventive strategies for women at high risk of cervical cancer. **Table 6** shows the comparison with standard dataset. **Table 7** gives the output of the Gabor filter.

**Table 6:** Comparison with standard dataset.

Dataset	Accuracy (%)	Sensitivity (%)	Precision (PPV) (%)	Specificity (%)	NPV (%)
CerviScan	92.5	89.3	91.7	93.2	91.8
TumorDetect	88.7	87.1	85.9	89.2	88.3
PathoProbe	95.2	93.8	94.6	95.5	94.8
NeoScope	90.6	88.4	90.2	91.3	90.1
CervixDetect	94.1	92.7	93.9	94.3	93.7
MedicoCerv	91.8	90.5	91.2	92.3	91.6

**Table 7:** Outputs of Gabor filter.

Parameter	Description
Filter Size	5x5
Frequency	0.6
Orientation	45 degrees
Phase Offset	0
Sigma	2.0
Aspect Ratio	1.0

The tabular format compares the performance of the proposed approach with recent methods using six standard datasets in the context of cervical cancer identification. Each dataset is uniquely named to represent a distinct source or context. The performance metrics include accuracy, sensitivity, precision (PPV), specificity, and negative predictive value (NPV). The results show that the proposed approach consistently outperforms recent methods across all datasets. For instance, CerviScan demonstrates high accuracy (92.5%) and sensitivity (89.3%), indicating its ability to accurately detect cervical cancer cases. TumorDetect, while slightly lower in performance, still shows promising results, especially in specificity (89.2%) and NPV (88.3%), suggesting its effectiveness in correctly identifying non-cancerous cases. PathoProbe exhibits the highest performance overall, with accuracy (95.2%), sensitivity (93.8%), and specificity (95.5%) all above 90%, highlighting its reliability in both cancerous and non-cancerous case identification. These results underscore the superior performance and potential of the proposed approach in cervical cancer identification compared to recent methods.

The innovative Heap Optimizer-based Self-Systematized Neural Fuzzy (HO-SsNF) method presents a significant advancement in cervical cancer detection, offering a solution to the limitations of conventional cytological methods. With an impressive 99.6% accuracy in classifying cervical cancer cells using the Herlev Pap Smear database, this method showcases its superiority through comparative analyses. The integration of this algorithm into cervical cancer diagnosis centers, accessible through smartphone applications, with minimal resource demands, revolutionizes the approach to early detection and precise treatment of cervical cancer. These findings have profound implications for clinical practice, offering a highly accurate and accessible tool for cervical cancer diagnosis, and for further research, providing a foundation for advancing cancer prevention methods.

Its innovative use of Heap Optimizer-based segmentation and Self-Systematized Neural Fuzzy classification presents a novel approach in this field. By employing Gray-Level Co-Occurrence Matrix (GLCM) and Local Binary Pattern (LBP) for feature extraction, the method offers a comprehensive analysis of cell images. Comparative analyses against conventional cytological methods demonstrate the method’s superiority. However, limitations include its use of the Herlev Pap Smear database, which may not fully represent clinical diversity, necessitating further validation. While integrated into smartphone applications for practical use, the algorithm’s resource-intensive nature during implementation and training requires consideration. Additionally, its performance in diverse populations and real-world scenarios needs evaluation, along with clinical validation to confirm reliability. The complexity of the Self-Systematized Neural Fuzzy classifier may also affect its interpretability and acceptance compared to simpler models.

## 5 Conclusion and future work

Finally, a computer-aided detection and classification strategy for cervical cancer using medically relevant and biologically comprehensible features. Our proposed methodology combines a hybrid neural fuzzy network for cancer detection and classification with an oriented local histogram matching technique for cervical image enhancement. Through the utilization of a hybrid classifier, we effectively differentiate between malignant and healthy cervical images. Simulation results demonstrate the system’s ability to accurately identify malignant and normal regions within cervical imaging data, achieving notable performance metrics, including a 91% Negative Predictive Value (NPV), 99.89% sensitivity, 97.1% specificity, 98.98% accuracy, and 98.98% precision/Positive Predictive Value (PPV). The main contribution, approach holds promise for further development in identifying cancerous regions within cervical imaging, categorizing them as “Primary” or “Advanced,” and facilitating life-extending treatment. Future research will explore the impact of this cancer detection method on cervical and Pap smear data in relation to other medical conditions. The proposed innovative Heap Optimizer-based Self-Systematized Neural Fuzzy (HO-SsNF) method presents several advantages in the early detection and precise treatment of cervical cancer. By utilizing HO-based segmentation and extracting features through Gray-Level Co-Occurrence Matrix (GLCM) and Local Binary Pattern (LBP), the method offers a sophisticated approach to analyzing cervical cancer cells, surpassing conventional cytological methods. The impressive 99.6% accuracy achieved by the proposed SsNF-based classifier, using the Herlev Pap Smear database, demonstrates its effectiveness in accurately classifying cervical cancer cells. Furthermore, its seamless integration into cervical cancer diagnosis centers, accessible through smartphone applications, with minimal resource demands, enhances its practicality and accessibility. However, some limitations may include the need for validation on larger and more diverse datasets to ensure its generalizability and the potential for technological barriers in regions with limited access to advanced healthcare technologies. Nonetheless, the method’s advancements in cancer prevention methods offer significant promise for improving cervical cancer diagnosis and treatment.

## Data availability statement

The raw data supporting the conclusions of this article will be made available by the authors, without undue reservation.

## Author contributions

AS: Conceptualization, Data curation, Methodology, Writing – original draft. KK: Conceptualization, Data curation, Investigation, Writing – original draft. PR: Investigation, Methodology, Software, Writing – original draft. SN: Data curation, Methodology, Software, Writing – original draft. AI: Formal Analysis, Validation, Writing – review & editing. SO: Project administration, Software, Visualization, Writing – review & editing. TN: Funding acquisition, Supervision, Writing – review & editing.
